# The effect of vitamin D supplementation on the size of uterine leiomyoma in women with vitamin D deficiency

**DOI:** 10.22088/cjim.10.2.125

**Published:** 2019

**Authors:** Maryam Hajhashemi, Maryam Ansari, Fedyeh Haghollahi, Bita Eslami

**Affiliations:** 1 Department of Obstetrics and Gynecology, Faculty of Medicine, Isfahan University of Medical Sciences, Isfahan, Iran; 2 Student Research Committee, Faculty of Medicine, Isfahan University of Medical Sciences, Isfahan, Iran; 3 Vali ASR Reproductive Health Research Center, Tehran University of Medical Sciences, Tehran, Iran; 4 Breast Disease Research Center (BDRC), Tehran University of Medical Sciences, Tehran, Iran

**Keywords:** Uterine leiomyomas, 25-hydroxyvitamin D_3_, Vitamin D deficiency

## Abstract

**Background:**

Uterine leiomyomas (fibroids) are the most common benign pelvic tumors in women of reproductive age with an incidence ranging from 5.4% to 77%, Also, there is a high prevalence of vit D deficiency in Iran and there are the numbers of in vivo and vitro biological studies on the relationship of vitamin D and uterine leiomyomas^.^ The aim of this study was to evaluate the effect of vit D supplementation on the size of uterine leiomyoma in women with vit D deficiency.

**Methods:**

This double-blind prospective clinical trial was performed on 69 patients with uterine leiomyomas who had vit D deficiency. Group A (n=35) was treated with vit D 50,000 IU every 2 weeks for 10 weeks, while group B (n=34) received placebo with same color and shape. Finally, the leiomyoma size in both groups was compared (IRCT: 20160521027998N5).

**Results:**

After a 10-week intervention, 25-hydroxyvitamin D_3_ levels were significantly higher in group receiving vitamin D (36.08 vs 16.25 ng/ml). (P<0.001) Leiomyomas size in vit D group significantly decreased as compared to placebo group (52.58 vs 61.11 mm, respectively).

**Conclusion:**

Our results showed that administration of vit D3 may reduce the size of leiomyoma. It seems that vitamin D administration is the effective way to treat leiomyoma.

Uterine leiomyomas (fibroids) are the most common benign pelvic tumors in women of reproductive age with an incidence ranging from 5.4% to 77%, which grow in the uterine muscle of premenopausal women (1, 2). African American race, nulliparity, obesity, and a positive family history of fibroids are the risk factors for high rate of leiomyoma (3, 4). They may be asymptomatic or can cause abnormal bleeding, pelvic pressure symptoms, infertility and growth or regress throughout the life. Leiomyoma affects millions of women and it is a leading cause of hysterectomy (5). Treatment includes medical therapy, surgical intervention, and uterine artery embolization or ablative techniques and depends on the patient's age, reason for treatment, concern of fertility preservation, and patient's preference (6-8). Some women do not want the traditional management of symptomatic fibroids such as surgery (hysterectomy or myomectomy) and wish to conserve their uterus and fertility (9). So, it seems that the side effect of less dangerous treatments and intervention should be considered. The nutrition role in the prevalence of leiomyoma was focused in some studies. Intake of dairy products, omega-3 fatty acid, soybean milk, food additives and sweeteners increase the risk of leiomyoma, but high fruit intake may reduce the leiomyoma risk in black women (10-13).

Some studies have recently shown that vit D deficiency is an important risk factor for uterine fibroids (14-16) and uterine fibroids express lower levels of vit D receptor (VDR) compared with myometrium (17). Vit D deficiency is 10 times more among African American women (40-45%) than the other people (4%) and also, leiomyoma in African American women is three times more prevalent than white women (18). As regards that the 1, 25(OH) 2D3 functions as a potent antiestrogen/antiprogesteronic agent, it may have utility as a novel therapeutic option for uterine fibroids (19).

There is a high rate of vit D deficiency among women especially in Iran. In a study performed among 1210 males and females in Tehran (2004), the prevalence of deficiency (vit D <35 nmol/L) was 81.1%20. Also, the high prevalence of hypo vitaminosis D was detected in a research commenced among 5232 men and women in five large cities of Iran with different weather conditions (20, 21).

Also, in vivo and in vitro studies showed that vitamin D inhibited the uterine fibroid growth as a potent anti-tumor agent (22, 23). According to the recommendations of the Endocrine Society, vit D status should be evaluated in patients with vit D deficiency (24) and in cases of vit D deficiency; 25-hydroxyvit D3 is more effective than sun exposure in increasing 25-hydroxyvitamin D3 (25).

Since, there is a high prevalence of vit D deficiency in Iran and also, there are numbers of in vivo and vitro biological studies on the relationship of vit D and uterine leiomyomas (14-16, 26-28). But the present data are insufficient to detect the role of vit D as a medical therapy for the treatment of uterine fibroids in humans, which would require a randomized controlled trial. So, we aimed to evaluate the effect of vit D supplementation on the size of uterine leiomyomas in Iranian women.

## Methods

**Study design and target group: **This double-blind clinical trial was conducted in Obstetrics and Gynecology Department of Isfahan Alzahra and Shahid Beheshti Hospitals, Center of Iran from August 2014 to December 2015. In this study, we used restricted randomization by random allocation rule and we used sequentially numbered, sealed, opaque envelope. These envelopes were prepared by main investigator and a trained midwife who selected an envelope for each patient was not aware of the content inside the envelope. Meanwhile, sonolgist who evaluated the size of myoma was not aware of patient's allocation. Finally, data analysis was conducted by a blind person, too (IRCT: 20160521027998N5).

 Based on randomized blocks with size 4; the first sample was assigned to group A, the second sample was included in group A, the third and the fourth samples were placed in group B, and such a random division continued in the following order until all 70 samples were divided.

Healthcare therapists and staff as well as the subjects were not aware of the groups assigned and the trained midwife in the clinic was only given specific codes which were entirely blind. (AABB, ABBA, ABAB, BBAA, BABA, BAAB..a.). Since the women who had myoma with 20-80 mm size were included in this study, we estimated the mean size of myoma will be about 50 mm. We estimated the size of myoma in vit D group that will be reduced by about 10 mm. By considering 15 mm standard deviation, we calculated that 36 samples would be required in each group with a power of 80% and α=0.05.

The leiomyoma size of patients receiving vit D (intervention group) was compared to patients in control group. Inclusion criteria consisted of patients referred to Obstetrics and Gynecology department of Alzahra and Shahid Beheshti hospitals with a diagnosis of leiomyomas, signed a consent form to participate in the study plus having the age between 35 -– 49 years, with 1 to 2 uterine leiomyomas size 20-80 mm and with serum levels of 25-hydroxyvitamin D_3_ <20 ng/ml. Exclusion criteria consisted of age <35 years or >49 years, consuming hormonal drugs, massive vaginal bleeding, sonographic evaluation of the characteristics of the uterus is difficult or in the presence of suspected adenomyosis. Furthermore, patients reporting malignancy, hypertension, diabetes, multiple sclerosis, autoimmune disorders, and coronary, hepatic, or renal diseases, pregnancy, menopause, hormonal treatment (including oral contraceptive) during the past 3 months, vit D supplementation, and chronic assumption of medications, dissatisfaction to continue participation in study, improper use of vit D and losing to follow-up due to various causes.

 The primary outcome included the size of the myoma and the level of vitamin D, and the secondary outcome including the symptoms of uterine myomas such as vaginal bleeding, pelvic pain, and urinary symptoms.

**Figure 1:**
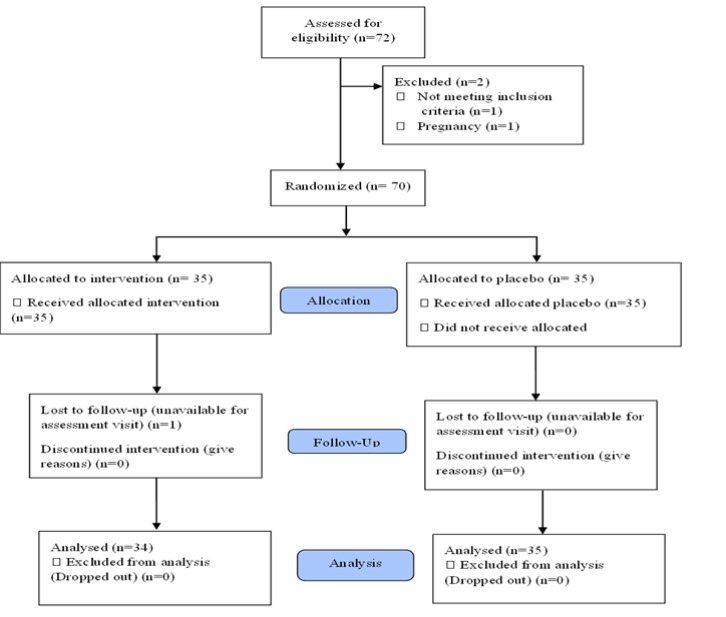
Study flowchart (CONSORT format)

**Participants: **The study flowchart is shown in figure 1. Seventy patients with a diagnosis of uterine leiomyomas, who had been diagnosed by obstetricians and based on inclusion and exclusion criteria, were included. Leiomyomas were diagnosed with transvaginal and transabdominal ultrasound screening (transabdominal allows for visualization of pedunculated leiomyomas that are not likely to be seen transvaginal). Masses of 2-8 cm in diameter and larger qualified as leiomyomas. Experienced sonographers were trained on the study protocol and worked under the direct supervision of an expertise sonologist. 

Sampling was performed in specific season and the amount of daily activity and exposure to sunlight in 9-12 every morning in both groups without the use of face and hand sunscreens during the sun exposure. 25-hydroxyvitamin D_3_ levels were measured in all eligible patients before intervention and in those with levels less than 20 ngr/ml (or 50 nmal/lit) were randomly divided into two groups using a block randomization procedure with matched subjects in each block based on daily activity and exposure to sunlight. Women were randomly assigned to receive vitamin D 50,000 IU every 2 weeks for 10 weeks (Iranian Zahravi Corp.) (n=35, Group A), while group B (n=35) received placebo consisting of exepian without vit D3 with the same color and shape (Zahravi co, Iran) and were given in the same paper bags as blinded controls. All participants, investigators and staffs were blinded to the treatment assignment during the whole period of study. For assessing compliance, patients were requested to return unused capsules at the follow-up visit. We used stored plasma samples to measure the circulating metabolite, 25-hydroxyvitamin D (25(OH) D), which is the accepted biomarker for vit D (19). Radioimmunoassay (RIA) for 25(OH) D (20) was conducted by specific laboratory for all patients. Sixty-nine patients completed the study; 34 from intervention group and 35 from control group. The study received ethics approval from the Ethics Committee of Isfahan University of Medical Sciences (394453), and all participants gave written informed consent. At the end of the study, 25-hydroxyvitamin D_3_ levels were measured and intrauterine ultrasound was performed to evaluate uterine leiomyoma size with same operator and in same previous center 6 months later. 

**Data analysis: **Data were analyzed and reported only for patients who completed the trial. Statistical analysis of data was performed using SPSS Version 22 software. The normal distribution of all studied parameters was checked with Kolmogorov-Smirnov test and we found that the distribution of the outcome variable was normal. Student t-test and paired t-test were used for variables which were distributed in a normal way. The two tailed p-value less than 0.05 was considered significant. A p-value <0.05 was considered significant. 

## Results

Demographic features in terms of age (P=0.592), BMI (P=0.841) and age at menarche (P=0.731) in both groups were similar. As well, the physical activity (P=0.976) and sun light exposure (P=0.645) were similar (table 1). Three patients were dropped out (n=2, no criteria and n=1 lost to follow-up) and finally, 69 patients completed the study. Before the intervention, studied variables did not show significant differences between the groups. 25-hydroxyvitamin D_3_ levels in intervention and placebo group were 16.82 ng/ml and 16.77 ng/ml, respectively (P=0.925) and the size of leiomyomas was 59.73 mm and 60.31 mm, respectively (P=0.847). As obtained, after a 10- week intervention, 25-hydroxyvitamin D_3_ levels were significantly higher in group receiving vit D as compared to placebo group (36.08 vs 16.25 ngr/ml) (P<0.001). Moreover, leiomyomas size significantly changed in vit D group. Leiomyomas, size in vit D group significantly decreased as compared to placebo group (52.58 vs 61.11 mm, respectively) (P=0.006), while by analyzing data with paired t test, the size of leiomyomas in control group did not change significantly (P=0.315).

**Table 1:** Studied variables before and after intervention in both control and vitamin D groups

**Group** **Variables**	**Vitamin D** **( group A)**	**Control** **( group B)**	**P-value**
**Studied variables 6 months before intervention in both control and vitamin D groups**			
Age (year)	40.58±4.26	40.6±4.08	0.592
BMI (kg/m2)	23.38±4.33	23.17±4.36	0.841
Age at menarche (years)	11.55±1.94	11.4±1.88	0.731
Sunlight exposure (min)	74.85±24.16	77.42±21.97	0.645
25-hydroxyvitamin D_3_ levels (ng/ml)	16.82±2.22	16.77±2.32	0.925
Leiomyoma size (mm)	59.73±13.51	60.31±11.3	0.847
**Studied variables 6 months after intervention in both control and vitamin D groups**			
25-hydroxyvitamin D_3_ levels (ng/ml)	36.08±2.83	16.25±2.24	<0.001
Leiomyoma size (mm)	52.58±13.72	61.11±11.16	0.006

* Independent T-Test

**Table 2:** Mean differences in studied variables in intervention and control group

**Variables**	**Mean**	**SD**	**95% Confidence Interval of the Difference**		**t**	**df**	**Sig.**
			**Lower**	**Upper**			
**Mean differences in studied variables in intervention group**							
25-hydroxyvitamin D_3_ levels (ng/ml)	-19.26	3.85	-20.61	-17.91	-29.133	33	<0.001
Leiomyomas size (mm)	7.14	3.86	5.79	8.49	10.79	33	<0.001
**Mean differences in studied variables in control group**							
25-hydroxyvitamin D_3_ levels (ng/ml)	-1.02	6.2	-3.06	1.01	-1.019	37	0.315
Leiomyoma size (mm)	-0.28	1.98	-0.94	0.36	-0.899	37	0.375

Paired T-test

## Discussion

This study aimed to evaluate the effect of vitamin D supplementation on the size of uterine leiomyomas in Iranian women. Our results showed that vit D supplementation has significant effect on the size of leiomyomas in premenopausal women with vit D deficiency. In other words, by the administration of vit D in patients in intervention group, the six of leiomyomas decreased by 7.14 mm. By comparing the size of leiomyomas between the groups (vit D and control), we observed significant reduction in intervention group. Besides, vit D administration increased the 25-hydroxyvitamin D3 levels obviously. In this regard, Halder SK et al. showed lower serum levels of either 25-hydroxyvitamin D3 or 1, 25-dihydroxyvitamin D3, related to increase the risk of symptomatic uterine fibroids (23).

 And also, in another study Halder SK et al, suggest that supplementation with the paricalcitol may be noninvasive medical treatment choice for uterine fibroids (29). Sharan C et al. and Bla¨uer M et al. have also recently reported that, 25-dihydroxyvitamin D3 induced leiomyoma growth inhibition via catechol-omethyl transferase in vitro (30, 31). The results of Halder SK study demonstrated that 1; 25-dihydroxyvitamin D3 treatment decreases the uterine leiomyoma tumor size in the Eker rat model (23). Most of the study demonstrated the effect of 1, 25-dihydroxyvitamin D3 on apoptosis, modulation of several cell growth genes, protein synthesis, and cell proliferation (23, 30-33). These functions are the base of anti-tumor effect of 1, 25-dihydroxyvitamin D3 on leiomyoma. Holder et al. in their study showed that estrogen and progesterone receptor on rat uterine leiomyoma cells reduced by receiving the 0.5 microgram per Kg of 1, 25-dihydroxyvitamin D3(23). 

The significant role of vit D3 deficiency in the uterine fibroid development resulted from Soumia Brakta et al;s study in which vitamin D3 administration is an effective, safe, nonsurgical medical treatment option for the uterine fibroids treatments (28) and the sufficient dose of vit D is associated with a reduced risk of uterine fibroids (29). The results of this study are consistent with to other in- vivo and in-vitro studies in which vit D reduces the frequency and the size of leiomyomas (14-16, 22, 23, 26-31,). Therefore, the administration of Vit D is a low cost and estimating a yearly cost of maintenance therapy in the vit D deficiency context is approximately $32 (34, 35,36). 

The limitations of this study were the small sample size and does not evaluate the effect of different levels of vit D3 and estrogen and progesterone receptors, physical activity, smoking, parity on leiomyoma size, further studies with larger sample size, and as well as clinical trial studies are recommended. Furthermore, as the study was conducted in Asian women, it may not be applicable in women with other ethnicities. In addition, the exact mechanism for these findings needs to be further investigated to be able to explain the mechanism of the reduction of leiomyoma tumor in humans.

In conclusion, the present results showed that vit D administration reduces the size of uterine myoma, and no case of toxicity was observed. It seems that vit D administration is the effective way to treat leiomyoma. 
